# Eosinophilic solid and cystic renal cell carcinoma with *TSC2* mutation: a case report and literature review

**DOI:** 10.1186/s13000-023-01341-9

**Published:** 2023-04-25

**Authors:** Xin He, Ying Chen, Hao Tang, Yujuan Xu, Xingyan Zhu, Caihong Wang, Qiang Chen, Deyu Guo

**Affiliations:** 1 Department of Pathology, Guiqian International General Hospital, Guiyang City, Guizhou Province China; 2 Department of Radiology, Guiqian International General Hospital, Guiyang City, Guizhou Province China; 3 Department of Urology, Guiqian International General Hospital, Guiyang City, Guizhou Province China

**Keywords:** Case report, Renal cell carcinoma, Eosinophilic solid and cystic renal cell carcinoma, *TSC2*, Mutations, Misdiagnosis

## Abstract

**Background:**

Eosinophilic solid and cystic renal cell carcinoma (ESC-RCC) is an under-recognized, emerging new entity of sporadic renal neoplasms, which is listed as a rare type of renal cell carcinoma in the 2022 WHO renal tumor classification. It is easily misdiagnosed because its characteristics are insufficiently understood.

**Case presentation:**

We report one case of ESC-RCC, a 53-year-old female patient with a right kidney mass found during clinical examination. The patient experienced no discomforting symptoms. Computer-tomography imaging at our urinary department showed a round soft tissue density shadow around the right kidney. Microscopic examination revealed a tumor displaying a solid-cystic composition of eosinophilic cells with unique features, revealed by characteristic immunohistochemical markers (CK20-positive/CK7-negative), and a nonsense mutation in *TSC2*. Ten months after the renal tumor resection, the patient presented in good condition with no recurrence or metastasis.

**Conclusions:**

The distinct morphological, immunophenotypic, and molecular characteristics of ESC-RCC we describe here, based on our case and the relevant literature, highlight the key points of the pathological and differential diagnosis of this novel renal neoplasm. Our findings will therefore deepen our understanding of this novel renal neoplasm and help reduce misdiagnosis.

## Introduction

The 2022 World Health Organization (WHO) kidney tumor classification lists eosinophilic solid and cystic renal cell carcinoma (ESC-RCC) as a new and rare RCC type, with an incidence rate of approximately 0.2% [1–3]. Because ESC-RCC is an indolent neoplasm associated with good prognosis and low metastatic potential, it is important to distinguish it from other renal cell carcinomas. However, the incidence of ESC-RCC remains low and less than 60 cases have been reported in the literature to date. Insufficient understanding of its characteristics very often leads to misdiagnosis. Here we report the case of a 53-year-old female with ESC-RCC in the right kidney, and analyze and summarize clinical data, histopathological characteristics, immunohistochemistry, and molecular findings, to improve our understanding of the disease and reduce misdiagnosis.

## Case report

**Fig. 1:**
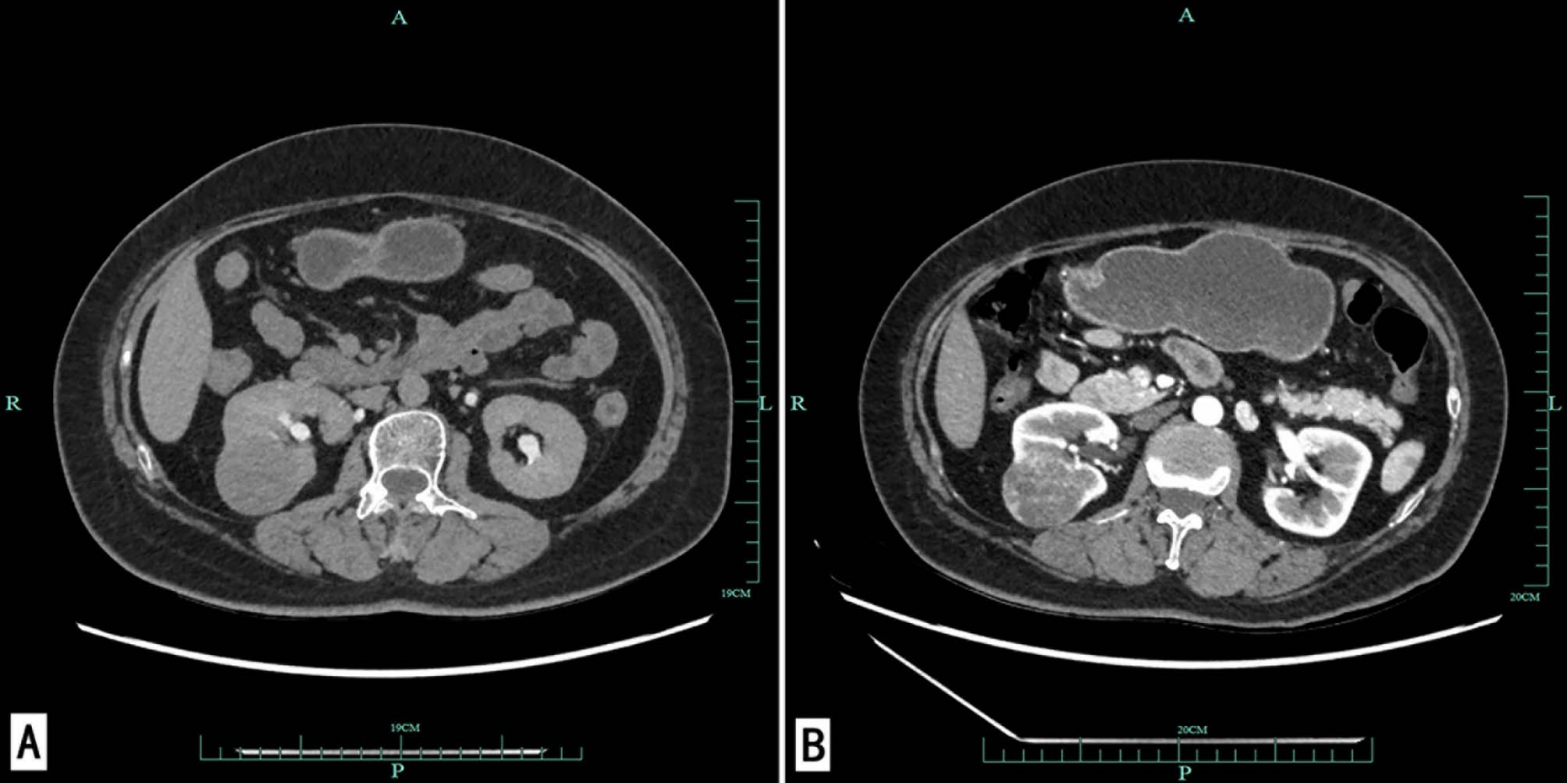
CT image of urinary system: A, the plain scan image showed a round soft tissue density shadow in the right kidney B, the enhanced image showed uneven and obvious enhancement in the cortical phase, and the degree of enhancement was slightly lower than that of the renal cortex

A 53-year-old female patient was hospitalized after a right renal mass was detected on clinical examination. The patient had not reported discomforting symptoms and had no relevant family history. Computer-tomography imaging at our urinary department showed a round soft tissue density shadow around the right kidney. The tumor size was approximately 41 mm × 40 mm, and its intensity appeared slightly lower than that of the renal cortex, which is a feature of malignant renal tumors (Fig. 1). The patient showed no obvious abnormalities in liver or kidney function or serum tumor markers. She underwent right renal tumor resection via laparoscopic robot-assisted surgery, and part of the excised kidney and tumor tissues were sent for pathological examination.

**Fig. 2:**
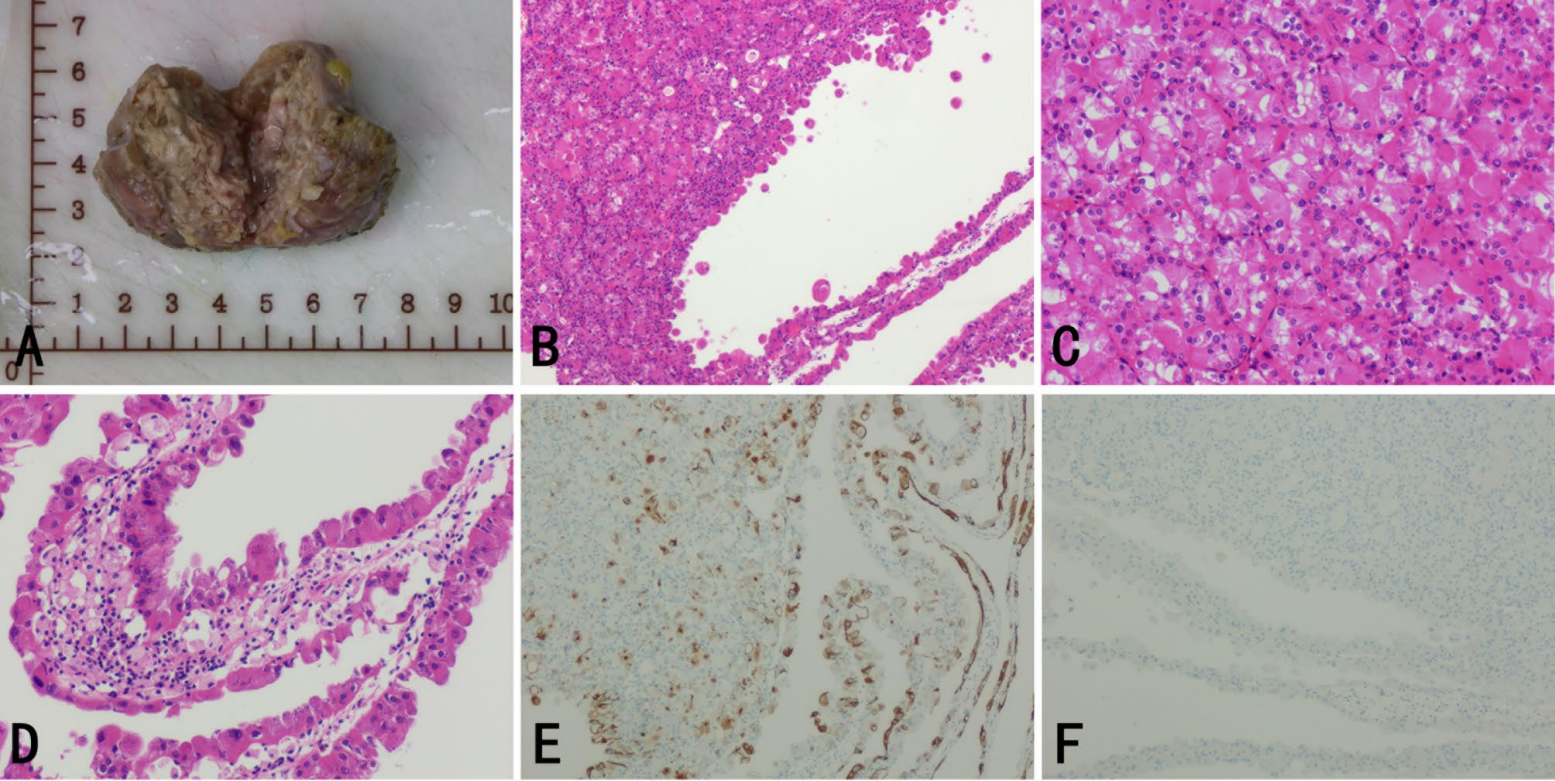
Pathological characteristics of the tumor: A, The gross tumor showed Clear boundaries on cut surface; B, The tumor showed solid and cystic structures, tumor cells were rich in eosinophilic cytoplasm. HE staining with magnification ×40; C, Solid areas: The tumor cells were arranged in a nest and sheet. HE staining with magnification ×100; D, Cystic areas: The tumor cells were arranged in hobnail-like, Foam cells and lymphocytes infiltration were observed in the stroma. HE staining with magnification × 100; E, The tumor cells were focal positive expression of CK20. Staining by the En Vision method with magnification ×40; F, The tumor cells were negative of CK7. En Vision method with magnification × 40

The gross mass with a grey-white cystic solid was 41 mm × 40 mm in size and clearly distinguishable from the surrounding renal tissue. Microscopic observation revealed that the cytoplasm of the tumor cells was rich in eosinophilic particles. Nuclei were round or oval in shape. Clear nucleoli were visible at 400× magnification in some cells. The nuclei had a WHO/ISUP grade 2. Cells were arranged cystically with hobnail cells lining the cystic wall in the cystic area. In the solid area, cells were arranged in nests and sheets. Eosinophilic bodies were visible in the cytoplasm, and focal foam cells and lymphocytes infiltration were observed in the stroma (Fig. 2). Immunohistochemical labeling indicated that pan-cytokeratin, vimentin, CK20, PAX-8, AMACR, and SDHB were positively expressed, to varying degrees. The expression of CD117, CK7, CD10, CA IX, and TFE3 was negative (Fig. 2). Next-generation sequencing (NGS) revealed a nonsense mutation with amino acid variant information c.1288 A > T p.R430 * in *TSC*, located in exon 13 of the gene and resulting in the termination of the encoded protein at arginine at position 430. The mutation-to-copy number ratio was 35.21%. Three additional missense mutations (*TSC2*, *CDK12* and *ERBB4*) were detected.

The tumor was solitary, 41 mm in diameter, and had clear boundaries. Microscopy showed visible cystic and solid areas. The tumor cells were rich in eosinophilic cytoplasm in the solid areas and in foam cells and lymphocytes in the stroma. As the patient showed the most important diagnostic characteristics of immunohistochemical markers (CK20-positive/CK7-negative) and a nonsense mutation in *TSC2*, we made the final diagnosis of ESC-RCC. At a followup examination 10 months after the surgery, we found no evidence of recurrence or metastasis.

## Discussion

ESC-RCC is a newly identified renal epithelial tumor with unique clinical manifestations, histopathological features, and a molecular immune phenotype. It is officially listed as a new RCC type in the WHO classification of renal tumors published in 2022. In the following, we summarize and analyze the relevant literature and discuss the clinical manifestations, histopathology, molecular immunohistochemical characteristics, clinical treatment, and prognosis of this novel renal neoplasm in relation to the case presented above.

### Clinical manifestations

In 2010, Schreiner et al. [4] first reported that ESC-RCC occurs almost exclusively in adult women with tuberous sclerosis (TSC). The tumor was also identified by Trpkov et al. [1, 2] in sporadic patients with non-tuberous sclerosis. Recently, it was found that TSC and ESC-RCC do not commonly co-occur, and that only approximately 10% of patients with ESC-RCC have concurrent clinical symptoms of TSC [5, 6]. Onset age ranges widely, from 14 to 79 years, with an average of 57 years. Men can also develop the disease [7, 8]. Clinical manifestations do not include obvious symptoms, and the tumors are usually discovered accidentally, without patients experiencing low back pain or hematuria, and show indolent biological behavior [9]. Some reports have shown that this disease can also metastasize to the lungs and liver [10, 11]. In the largest series of 19 patients with ESC-RCC, with a mean follow-up of 49.6 months, 89% had stage pT1 disease. Among them, three died of other diseases [2]. The primary treatment for ESC-RCC is surgical resection with partial or radical nephrectomy, according to the tumor stage. The case presented here represents an adult female who was clinically asymptomatic during examination. Ten months after renal tumor resection, the patient was in good condition and showed no recurrence or metastasis.

### Pathological characteristics

#### Morphological characteristics

Typical morphological characteristics of ESC-RCC are that the tumor is mostly solitary, generally grayish-yellow, and 12–135 mm in diameter (average, 34 mm). The tumor is cystic and well demarcated from the surrounding renal tissue. Large or microcystic structures are observed in the cystic areas of the tumor, and foam cells and lymphocytes infiltration in the focal stroma. The tumor cells in solid areas are arranged in nest, acinar, and sheet structures, with rich eosinophilic cytoplasm and eosinophilic bodies. There are also reports of papillary, vacuolar structures and scattered single or tiny cell clusters [12]. In our case, ESC-RCC was a well-defined solitary tumor with a cystic section and a clear boundary. Microscopically, visible cystic and solid areas were observed. The cystic area differed in size and the cystic wall was lined with hobnail-like tumor cells. The tumor cells were rich in eosinophilic cytoplasm in the solid areas and foam cells and lymphocytes in the stroma, which is the typical pathological morphology of ESC-RCC.

#### Immunohistochemical and molecular characteristics

We detected varying expression levels of the immunohistochemical markers CK20, PAX-8, and AMACR in the tumor cells of our patient, but not of CD117, CK7, CD10, and CA IX. CK20-positivity/CK7-negativity is the most important diagnostic marker for ESC-RCC. Focal CK7-positivity has been reported in 25% of cases [1, 2, 13]. To assess the relevant molecular genetics of ESC-RCC, Trpkov et al. analyzed molecular karyotype expression in 12 ESC-RCC cases, and found that the tumor can exhibit copy number acquisition of chromosomes 16, 7, 13q, and 19p, copy number deletion of Xp11.21 and 22q11.23, and loss of heterozygosity of *TSC1* and *TSC2*. Multiple other studies have reported the presence of mutations in both *TSC1* and *TSC2* in ESC-RCC [8, 14, 15]. Although some patients do not exhibit clinical manifestations of TSC, they do show a loss of heterozygosity or *TSC2* copy number acquisition. However, the specific molecular genetic characteristics of ESC-RCC require further investigation. Although our patient exhibited no clinical manifestations of TSC, a nonsense mutation in *TSC2* was detected, alongside varying expression levels of CK20 and PAX-8 and no expression of CD117 and CK7 on immunohistochemistry. These features supported the diagnosis of ESC-RCC.

#### Differential diagnosis

For an accurate diagnosis, ESC-RCC needs to be differentiated from other renal tumors with eosinophilic cytoplasm or cystic solid structures, including the following: (1) Clear cell renal cell carcinoma: The tumor cells are arranged in a nest or acinar structure, and the cytoplasm is eosinophilic. However, the stroma is rich in a thin parenchymal vascular network. It can also be distinguished by immunohistochemistry, with the markers CA IX, CD10, and PAX-8 showing mostly positive and CK7 mostly negative results. (2) Chromophobe renal cell carcinoma: The cytoplasm of chromophobe renal cell carcinoma is transparent or eosinophilic, and the tumor cells are solid or acinar, usually without large cystic structures, and contain thick wall blood vessels. The tumor cells are large and polygonal with clear cell membranes and visible perinuclear empty halos. The immunohistochemical marker CK7 and on Hale colloidal iron staining are a characteristic diffuse strongly positive expression. (3) Xp11.2 translocation/TFE3 gene fusion-associated renal cell carcinoma: Its microscopically visible rich cytoplasm is transparent or eosinophilic, with a broad papillary or, in rare cases, a cystic or microcystic structure, obvious nucleoli, and stroma with a large number of psammoma bodies. High expressed TFE3 fusion proteins are characteristic immunohistochemical markers. (4) Succinate dehydrogenase-deficient renal cell carcinoma: It is mainly manifested by the lack of SDHB protein expression and *SDH* gene germline mutation; the microscopic features of tumor cells are a lobulated contour, pushing growth, a solid nest arrangement, cystic structure, rich cytoplasm, eosinophilic flocculation, and vacuolar appearance. The absence of the expression of immunohistochemical marker SDHB is necessary for disease diagnosis. (5) Epithelioid angiomyolipoma: The tumor cells are mostly solid, composed of polygonoid epithelioid cells and spindle cells, cytosolic eosinophilic, and immunohistochemically expressing Melan-A and melanosome-associated antigen. However, cases of ESC-RCC with epithelioid angiomyolipoma characteristics have been reported, and the tumor cells express Melan-A, melanosome-associated antigen, CK20, and PAX8, among which CK20 and PAX8 may be effective for a differential diagnosis. Molecular detection methods can also be used when making a differential diagnosis is difficult.

## Conclusion

In conclusion, ESC-RCC is a new rare type of RCC, according to the 2022 edition of the WHO renal tumor phenotype, with unique clinical manifestations, pathological features, and molecular immunophenotypes. Here, we report a case of ESC-RCC characterized by eosinophilic cytoplasm with characteristic immunohistochemical markers (CK20-positive/CK7-negative) and a nonsense mutation in *TSC2*. Our analysis and summary of the clinical data, morphological characteristics, immunohistochemistry, and molecular findings of the disease will deepen our understanding of this novel renal neoplasm and help practitioners make pathological and differential diagnoses.

## Data Availability

All data generated or analyzed during the current study are included in this published article.

## Abbreviations

ESC-RCC: Eosinophilic solid and cystic renal cell carcinoma
TSC: Tuberous sclerosis
WHO: World Health Organization
